# Supplementary material for: MetaRibo-Seq measures translation in microbiomes
Source: Nat Commun. 2020 Jun 29;11:3268. doi: 10.1038/s41467-020-17081-z (PMC7324362; doi:10.1038/s41467-020-17081-z)
Supplement: Supplementary file 10 — Supplementary Data 7 [file 41467_2020_17081_MOESM10_ESM.zip › File2/Confidence_High_Taxonomy/34534_out.krona.html]

Javascript must be enabled to view this page.

members
magnitude
magnitudeUnassigned
count
unassigned
taxon
rank

34534\_out

173

2
173
superkingdom

976
1
phylum

1
class
117743

200644
1
order

49546
1
family

1
genus
1016


SRS893282\_contig\_number\_contig-100\_3932.36629
1
species
1017

159
phylum
32066

203490
159
class

203491
159
order

14
family
203492

1

SRS020334\_contig\_number\_8118
14
genus
848

851
species
8
1

SRS023930\_contig\_number\_contig-100\_1171.1171

76857
subspecies
6

SRS016474\_contig\_number\_211SRS024087\_contig\_number\_912SRS043239\_contig\_number\_290SRS043729\_contig\_number\_contig-100\_1472.38476SRS893232\_contig\_number\_7894SRS893282\_contig\_number\_contig-100\_465.466

155615
1
subspecies

SRS054430\_contig\_number\_6830


SRS021528\_contig\_number\_10584SRS042643\_contig\_number\_224SRS056461\_contig\_number\_1861SRS077283\_contig\_number\_11729SRS142680\_contig\_number\_6610
5
species
860

family
145
1129771

genus
143
32067

5
species
157688

SRS015755\_contig\_number\_contig-100\_1916.33777SRS019028\_contig\_number\_contig-100\_905.905SRS022083\_contig\_number\_32SRS077549\_contig\_number\_14948SRS144402\_contig\_number\_28


SRS017511\_contig\_number\_33SRS018975\_contig\_number\_602SRS019128\_contig\_number\_1101SRS019225\_contig\_number\_contig-100\_4400.32514SRS022281\_contig\_number\_9806SRS022980\_contig\_number\_contig-100\_18039.43615SRS023538\_contig\_number\_2SRS043755\_contig\_number\_1826SRS045763\_contig\_number\_contig-100\_4599.52176SRS056323\_contig\_number\_11471SRS075961\_contig\_number\_contig-100\_95.95SRS146773\_contig\_number\_14501
species
12
157692


SRS019607\_contig\_number\_13629SRS057124\_contig\_number\_676SRS058336\_contig\_number\_11351SRS077568\_contig\_number\_8654SRS1055073\_contig\_number\_60SRS144368\_contig\_number\_5662SRS148262\_contig\_number\_11189SRS893175\_contig\_number\_265
species
8
157687


SRS097912\_contig\_number\_388
species
1
1785996

35
species
712357

SRS015378\_contig\_number\_1498SRS015899\_contig\_number\_32SRS016092\_contig\_number\_contig-100\_1292.1292SRS016139\_contig\_number\_365SRS017025\_contig\_number\_1608SRS018061\_contig\_number\_contig-100\_1226.1227SRS018616\_contig\_number\_contig-100\_33.34SRS018665\_contig\_number\_20146SRS019077\_contig\_number\_8319SRS019129\_contig\_number\_227SRS022536\_contig\_number\_12212SRS042984\_contig\_number\_3663SRS050669\_contig\_number\_contig-100\_196.37647SRS050783\_contig\_number\_2044SRS051378\_contig\_number\_7742SRS053917\_contig\_number\_16944SRS055378\_contig\_number\_14985SRS055401\_contig\_number\_20SRS057931\_contig\_number\_contig-100\_1325.1326SRS062931\_contig\_number\_2288SRS063215\_contig\_number\_contig-100\_22789.61329SRS075876\_contig\_number\_3799SRS097871\_contig\_number\_contig-100\_14.14SRS098057\_contig\_number\_2642SRS098624\_contig\_number\_contig-100\_1341.57716SRS104539\_contig\_number\_1031SRS104968\_contig\_number\_302SRS105121\_contig\_number\_33SRS143360\_contig\_number\_contig-100\_978.979SRS144124\_contig\_number\_contig-100\_233.38577SRS144707\_contig\_number\_contig-100\_54.55SRS147442\_contig\_number\_25SRS893233\_contig\_number\_contig-100\_255.256SRS893328\_contig\_number\_2540SRS893376\_contig\_number\_contig-100\_3006.3007


SRS015590\_contig\_number\_139SRS015644\_contig\_number\_1774SRS015797\_contig\_number\_108SRS016647\_contig\_number\_126SRS017383\_contig\_number\_contig-100\_6363.6364SRS017533\_contig\_number\_8742SRS017751\_contig\_number\_2028SRS018145\_contig\_number\_3835SRS018145\_contig\_number\_contig-100\_341.341SRS018157\_contig\_number\_1326SRS018439\_contig\_number\_12507SRS018875\_contig\_number\_contig-100\_570.41118SRS018923\_contig\_number\_748SRS018969\_contig\_number\_6573SRS019022\_contig\_number\_10937SRS019126\_contig\_number\_733SRS019327\_contig\_number\_897SRS020222\_contig\_number\_contig-100\_1422.1422SRS020540\_contig\_number\_290SRS020628\_contig\_number\_21399SRS022464\_contig\_number\_4068SRS023617\_contig\_number\_623SRS023958\_contig\_number\_10277SRS023964\_contig\_number\_10231SRS024277\_contig\_number\_2391SRS043461\_contig\_number\_491SRS047210\_contig\_number\_12402SRS048317\_contig\_number\_83SRS049448\_contig\_number\_contig-100\_611.612SRS052227\_contig\_number\_contig-100\_360.360.360SRS054569\_contig\_number\_28SRS057205\_contig\_number\_contig-100\_1291.36071SRS057355\_contig\_number\_172SRS057692\_contig\_number\_contig-100\_343.41633SRS063193\_contig\_number\_298SRS063373\_contig\_number\_1122SRS063373\_contig\_number\_contig-100\_467.36423SRS064022\_contig\_number\_12SRS064423\_contig\_number\_contig-100\_5722.40147SRS065299\_contig\_number\_33SRS075084\_contig\_number\_10441SRS075927\_contig\_number\_259SRS075957\_contig\_number\_contig-100\_1376.1376SRS077016\_contig\_number\_88SRS077327\_contig\_number\_525SRS077736\_contig\_number\_1446SRS078775\_contig\_number\_7720SRS097895\_contig\_number\_contig-100\_680.680SRS097968\_contig\_number\_contig-100\_491.34723SRS098065\_contig\_number\_2367SRS098616\_contig\_number\_1639SRS098818\_contig\_number\_2189SRS102808\_contig\_number\_contig-100\_1711.1711SRS103971\_contig\_number\_10823SRS105068\_contig\_number\_146SRS105068\_contig\_number\_10597SRS1054881\_contig\_number\_contig-100\_106.107SRS1055063\_contig\_number\_contig-100\_9405.43801SRS1055071\_contig\_number\_contig-100\_202.203SRS143039\_contig\_number\_contig-100\_8934.8935SRS143088\_contig\_number\_8222SRS143529\_contig\_number\_12325SRS144014\_contig\_number\_2287SRS144424\_contig\_number\_contig-100\_465.465SRS146738\_contig\_number\_1456SRS146996\_contig\_number\_8362SRS147088\_contig\_number\_contig-100\_239.239SRS147149\_contig\_number\_282SRS147380\_contig\_number\_464SRS148284\_contig\_number\_5750SRS148514\_contig\_number\_35SRS149579\_contig\_number\_2440SRS149690\_contig\_number\_contig-100\_2436.2437SRS149882\_contig\_number\_143SRS149938\_contig\_number\_contig-100\_2400.2400SRS149961\_contig\_number\_contig-100\_209.209SRS893173\_contig\_number\_8759SRS893173\_contig\_number\_contig-100\_108.109SRS893274\_contig\_number\_9759SRS893277\_contig\_number\_contig-100\_2963.84868SRS893359\_contig\_number\_984
712359
species
81


SRS142664\_contig\_number\_13748
species
1
671213

2
genus
168808

187101
2
species

SRS023466\_contig\_number\_contig-100\_1962.1962SRS023604\_contig\_number\_contig-100\_4141.4141

2
phylum
1224

1
class
28211

204441
order
1

family
1
41295

191
genus
1

species
1
1262705

SRS013965\_contig\_number\_203

28216
class
1

order
1
80840

80864
family
1

genus
1
219181

species
1
219182

SRS142551\_contig\_number\_2009

phylum
1
544448

class
1
31969

order
1
2085

2092
family
1

2129
1
genus

species
1
134821

SRS146924\_contig\_number\_contig-100\_964.964

95818
1
phylum


SRS056622\_contig\_number\_1245
447454
species
1

phylum
2
201174

1760
1
class

order
1
2037

1
family
2049

1654
genus
1


SRS017007\_contig\_number\_1042
55565
species
1

class
1
84998

84999
1
order

family
1
1643824

1380
1
genus


SRS051791\_contig\_number\_contig-100\_4367.33412
1382
1
species

7
phylum
1239

3
class
909932

1843489
order
1

31977
1
family

genus
1
29465


SRS893339\_contig\_number\_25
29466
1
species

order
2
909929

family
2
1843491

970
genus
2

1
species
712538

SRS056327\_contig\_number\_148


SRS019129\_contig\_number\_contig-100\_4925.85142
1
species
135083

4
class
91061

3
order
1385

1378
3
genus

1
species
84135

SRS016002\_contig\_number\_17295

1
species
29391

SRS077022\_contig\_number\_5532


SRS147122\_contig\_number\_contig-100\_257.257
species
1
1379

186826
1
order

1
family
186827

46123
1
genus


SRS075959\_contig\_number\_contig-100\_639.55876
species
1
46125
